# Research Methods and Progress of Patellofemoral Joint Kinematics: A Review

**DOI:** 10.1155/2019/9159267

**Published:** 2019-03-24

**Authors:** Zhenguo Yu, Jie Yao, Xingliang Wang, Xing Xin, Ke Zhang, Hong Cai, Yubo Fan, Bin Yang

**Affiliations:** ^1^Orthopedics Department, Peking University Third Hospital, 49 Huayuanbei Road, Haidian District, Beijing, China; ^2^Key Laboratory for Biomechanics and Mechanobiology of Ministry of Education, School of Biological Science and Medical Engineering, Beijing Advanced Innovation Centre for Biomedical Engineering, Beihang University, 37 Xueyuan Road, Haidian District, Beijing, China; ^3^Orthopedics Department, No. 101 Hospital of PLA, Xingyuan North Road, Liangxi District, Wuxi, China; ^4^Orthopedics Department, Peking University International Hospital, Life Park 1, Zhongguancun Life Science Park, Changping District, Beijing, China; ^5^National Research Center for Rehabilitation Technical Aids, Beijing, China

## Abstract

Patellofemoral pain syndrome has a high morbidity, and its pathology is closely associated with patellofemoral joint kinematics. A series of *in vivo* and *in vitro* studies have been conducted to explore patellofemoral kinematics, and the findings are relevant to the diagnosis, classification, and management of patellofemoral diseases and even the whole knee joint. However, no definite conclusion on normal patellofemoral kinematics has been established. In this study, the measurement methodologies of patellofemoral kinematics (including data collection methods, loading conditions, and coordinate system) as well as their advantages and limitations were reviewed. Motion characteristics of the patella were analyzed. During knee flexion, the patellar flexion angle lagged by 30–40% compared to the tibiofemoral joint flexion. The patella tilts, rotates, and shifts medially in the initial stage of knee flexion and subsequently tilts, rotates, and shifts laterally. The finite patellar helical axis fluctuates near the femoral transepicondylar axis or posterior condylar axis. Moreover, factors affecting kinematics, such as morphology of the trochlear groove, soft tissue balance, and tibiofemoral motion, were analyzed. At the initial period of flexion, soft tissues play a vital role in adjusting patellar tracking, and during further flexion, the status of the patella is determined by the morphology of the trochlear groove and patellar facet. Our findings could increase our understanding of patellofemoral kinematics and can help to guide the operation plan for patients with patellofemoral pain syndrome.

## 1. Introduction

Patellofemoral pain syndrome (PFPS) is associated with a high morbidity 13–20% [1–4] and a prevalence of up to 40% among athletes [5]. Morbidity in women is 2–4 times greater than that in men [1, 6]. A number of studies have shown a significant correlation between PFPS and several factors, including reduced strength of the quadriceps and reduced Q-angle, patellofemoral malalignment, dysplasia of the femoral trochlea, and patellofemoral disorders [7–11]. However, the pathogenesis of these factors and quantitative patellofemoral biomechanics remain unclear, which increases the difficulty in diagnosing, classifying, and remedying patellofemoral diseases.

The classification and severity of PFPS have been related to different types of patellofemoral kinematics. For instance, patients with patellar dislocation tend to complain of discomfort due to excessive lateral shift of the patella when the knee joint approaches full extension [12, 13]. An abnormality in the elasticity of iliotibial band tension is also regarded as a cause of PFPS [14], which can affect patellar rotation at deep knee flexion [15]. Exploring the patellofemoral biomechanics can contribute to a better understanding of various patellofemoral disorders and improve diagnostic and curative approaches. Nevertheless, no definite conclusion on the normal patellofemoral kinematics has been established.

**Figure 1:**
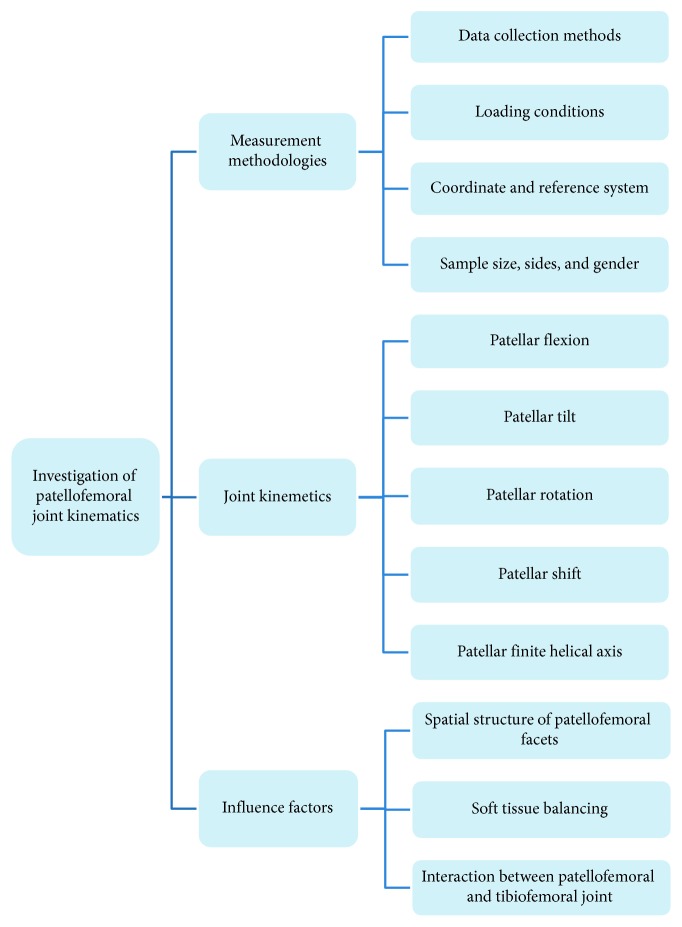
The overview schematic of this review. In this review, different measurement methodologies were reviewed, which often related to the description of patellar tracking. Then, the patellofemoral kinematics of previous studies was summarized and analyzed. The factors influencing the patellofemoral kinematics are also discussed.

Many researchers have undertaken *in vivo* and *in vitro* studies to clarify patellofemoral joint kinematics and their association with diseases, with a view to providing evidence necessary for determining a standard therapeutic regimen. However, joint loading conditions and coordinate system settings can affect the results of kinematic measurements. Consequently, the relevant studies have not reached consensus. Hence, in this study, different measurement methodologies were reviewed, which could affect the description of patellar tracking. Then, the patellofemoral kinematics of previous studies were summarized and analyzed. The factors influencing the patellofemoral kinematics are also discussed (Figure 1). Moreover, we identified the bottlenecks in patellofemoral kinematics research and proposed a methodology for further studies.

## 2. Measurement Methodologies

Researchers have attempted to measure patellar tracking using multiple approaches, each of which involves the following aspects: data collection methods, loading and boundary conditions, establishment of a coordinate system, and other factors including gender, side of knee (right or left), and sample size. Compared to *in vitro* measurement, *in vivo* measurement was usually noninvasive, yet its accuracy is relatively lower. Loading conditions could influence the patellar tracking. Different coordinate systems will lead to varying descriptions of the tracking.

### 2.1. Data Collection Methods

Imaging methods, including ultrasound [16–18], X-ray [19], computed tomography (CT) [20–24], single- and biplane fluoroscopy [25–27], and magnetic resonance (MR) imaging [28–35], have been used for *in vivo* measurements. With the advantages of noninvasive, radiation-free acquisition, and soft tissue contrast, MR imaging has been widely applied to the research on patellofemoral kinematics [28, 31, 32, 34, 35]. After the MR scannings of the joint, femur and patella were segmented and reconstructed to 3D models. A series of models at several different knee flexion angles were registered to a common coordinate system to depict patellar tracking quasistatically [28]. However, static scanning can only acquire finite patellofemoral images at a few specific knee flexion angles (usually 5 to 10 angles between 0° and 120°), based on which a consecutive patellar tracking is estimated with an interpolation algorithm. Therefore, the patellofemoral kinematics reestablished using static images cannot accurately reflect the dynamic motion of the joint [36]. Single- and biplane fluoroscopy techniques have shown 3D dynamic changes in real-time knee kinematics. However, the fluoroscopy techniques have a risk of radiation. Furthermore, single-plane fluoroscopy is prone to large error at the translation along the axis orthogonal to the image plane. Biplane fluoroscopy is not applicable for measuring patellofemoral kinematics after knee arthroplasty, because the patella will be obscured by the metal femur implant, and is only visible in a single plane (the sagittal plane) [27]. Studies based on CT allow the objective measurement of some tracking indices, such as lateral patellofemoral angle and patellofemoral congruence angle, which represent patellar tilt and shift. Patellar maltracking may appear at diverse ranges of knee flexion, yet the knee flexion angles of the previous studies were often less than 60° due to the limitation of the field of view in CT and MR machines. With the imaging devices being optimized, the knee flexion range captured in the recent studies has been increased to greater than 90° [31, 32], which could contribute to a more comprehensive understanding of patellofemoral joint biomechanics.

To address the aforementioned deficiencies of quasistatics and field of view limitations, motion capture methods have been used in the measurement of patellofemoral joint kinematics dynamically [15, 17, 37–44]. In motion capture measurement, the optical or electromagnetic sensors were fixed on patella. Since sensors' motion can be accurately collected, the patellar motion can be calculated according to sensors' motion. In order to obtain accurate joint motion, the sensor and the bone must be relatively immobile; so, it is often fixed on the bone with steel pins. However, earlier researchers have implanted metal probes into the patella, which are regarded as patellar markers [45]. Considering their invasiveness, most of these methods were applied in *in vitro* studies [15, 17, 39–44]. To obtain the *in vivo* joint motion noninvasively, some researchers used body surface markers to measure *in vivo* patellofemoral kinematics [37, 38]. Because of the relative movement between soft tissues and deeper bones during knee flexion, the errors of the measurement in three rotational and translational degrees of freedom increased with the knee flexion angle.

### 2.2. Loading Conditions

Loading conditions (e.g., weight-bearing loading and quadriceps force) could influence the results of patellar tracking measurements. Researches have explored the patellar tracking with different weight-bearing loadings [16, 22, 29–32, 37, 38, 46]. Non- and full-weight-bearing loadings could be achieved by sitting and standing. A loading device applying a compressive force at the foot was also designed to apply simulated partial weight-bearing conditions [46]. During complete knee extension, patellar tracking is significantly affected by weight-bearing conditions [47]; patellofemoral congruence angles are −6° (non-weight-bearing) and 12.8° (weight-bearing), which indicates that bearing could tilt and transfer the patella laterally [29]. To investigate the influence of muscle force on the patellar tracking, differences between active and passive knee extension-flexion were also quantified [29, 36]. These findings may provide insight into the causes of anterior knee pain and motion pain.

Researches have proven that magnitude and direction of muscle force affect patellar tracking [17, 29]. As quadriceps changed from relax to contraction at knee extension, the sulcus angles at the midpatella increased from 146° to 172°, which implied that the patella moved proximally [29]. The quadriceps force ranging from 10 to 175 N has been applied in most *in vitro* studies; quadriceps force of 175 N was commonly utilized since it extended the knee against gravity with the femur horizontal [17, 28, 36, 39–41, 43]. Lorenz et al. investigated the effect of quadriceps force direction on the patella position. As the force moved from vastus medialis to vastus lateralis, patella lateral rotation and lateral tilt increased [17]. Besides the quadriceps, iliotibial band tension also has an effect on patella tracking. Increasing iliotibial band tension will lead to an increase in patellar shift, tilt, and flexion angles at knee flexion between 60° and 75° and an increase in patellar rotation at knee flexion between 75° and 90° [15]. Furthermore, because of the difference in muscle movement during knee extension and during flexion, the patella tended to be closer to the distal and lateral femur during flexion than extension [39]. Therefore, subsequent studies of patellofemoral kinematics need to control the factors including muscle force magnitude and direction, as well as knee flexion or extension status.

In more complicated loading conditions related to daily activities, patellar tracking varies greatly with the motion patterns of the knee joint [33, 36]. Considering that symptoms resulting from patellar maltracking might occur at a particular moment of the gait cycle or in specific action modes [30, 36, 38], measurement techniques suitable for complex physiological activities need to be developed.

### 2.3. Coordinate System

Selecting different coordinate systems will have a significant impact on the description of the patellar tracking [47] and may yield varying conclusions. Coordinate systems based on the finite helical axis (FHA) and body-fixed axes are commonly applied for patellofemoral kinematics [48]. It is simple to depict the joint action by the helical axis. However, due to the atactic circular motion of the patella, an ever-changing helical axis is not applicable to clinical analysis. Coordinate systems based on body-fixed axes are much easier for physicians to understand [47].

Three primary coordinate systems have been involved in previous research: femoral coordinate system, patellar coordinate system, and femur-patella hybrid coordinate system [15, 17, 28, 31, 38, 42, 47] (Supplementary Material (available here)). A small number of studies considered the anatomical landmarks of the tibia for establishing the coordinate system [17, 35]. As patellofemoral kinematics refers to patellar movement relative to femur, descriptions of patellar tracking based on tibia appear unsatisfactory [36].

The three axes in the femoral coordinate system include the proximal-distal (PD) axis (longitudinal axis), medial-lateral (ML) axis, and anterior-posterior (AP) axis. The femoral anatomical and mechanical axes were often used to determine the longitudinal axis, and at times, a line from greater trochanter to the midpoint of the transepicondylar axis (TEA) is also considered [37, 44]. Moreover, the femoral groove axis has been used occasionally, despite the difficulty in fitting the curving trochlear groove into a straight line [47]. Because of less variation in the medial and lateral epicondyles, as well as the posterior condyles among different individuals, the TEA [17, 23, 30–32, 35, 37, 38, 40, 44] and posterior condylar line [32, 33, 36, 47] were often regarded as ML axis. The femoral AP axis can be established by the cross product of the longitudinal and ML axes [17, 19, 30]. Despite the extensive application of the femoral coordinate system, it still has some inextricable disadvantages. For instance, the definitions of patellar rotation and tilt will be confused with each other when knee flexion exceeds 90° [47]; hence, describing patellar rotation and tilt using the femoral axes at deep knee flexion is not appropriate.

Similar to the femoral coordinate system, the patellar coordinate system consists of three orthometric axes. However, to build up a patellar coordinate system is not easy as the patellae have irregular morphology and nondistinct anatomical structures. Li et al. [49] and Nha et al. [31] attempted to develop a fitting bounding box (FBB) around the patella so that it touched the superior-inferior, anterior-posterior, and medial-lateral borders of the patella. The center of the box was defined as the patellar center. The line along the superior-inferior direction was defined as PD axis of the patella [31]. In addition, Rainbow et al. [22] established a fictitious line as PD axis, which was parallel to the posterior vertical ridge (PVR) of the patella and intersected with the patellar center. The PD axes established by above two methods will have a 11.13° ± 4.1° difference [24]. According to this angle, the results based on two coordinate systems could be compared by coordinate transformation. Moreover, some scholars first determined the patellar center and then established the coordinate axes according to the femur coordinate system [50].

Various coordinate systems have been utilized in previous researches, but few studies reported the influence of the different systems on the measurement results. Bull et al. [47] revealed that the discrepancy between the patellar flexion angle around the femoral ML axis and that around the patellar ML axis reaches up to 26%, and a ten-fold difference in the patellar shift was observed (2.2 and 22 mm, respectively). Relative motion between coordinate systems could be a main cause of discrepancy. To make the description of the patellar tracking anatomically significant and reduce the error due to joint motion, the femur-patella hybrid coordinate system was proposed and gained significant popularity. In this system, Axis-1 is defined as the femoral ML axis, Axis-2 is defined as the patella PD axis and moved with the patella, and Axis-3 is defined as the axis perpendicular to Axis-1 and Axis-2 [31, 36, 38, 47]. The midpoint of the Axis-1 could be selected as the origin [30, 31]. Based on this hybrid coordinate system, patellar flexion is defined as the rotation of the patella around Axis-1 (femoral ML axis). Patellar tilt is defined as the rotation of the patella around Axis-2 (patella PD axis). Patellar rotation is defined as the rotation of the patella around Axis-3 (the floating axis). Patellar shift is defined as the movement of the patellar center along Axis-1. This joint coordinate system is easier to establish, and it decreases the mutual interference between different degrees of freedom (DOFs) of patellar movement. This is why it has been highly recommended in recent studies.

### 2.4. Other Factors

In studies on patellofemoral biomechanics, other factors including gender, side of knee (right of left), and sample size may play a role in the conclusion. Previous study indicated that although women are more prone to PFPS than men, patellar tracking does not show significant gender differences [51]. A reason for this might be that other factors such as hormones led to the women's susceptibility to PFPS. Studies on the side factor revealed that patellar movement was generally symmetrical between left and right knees, yet asymmetrical tracking still exist, especially in anterior-posterior and medial-lateral directions [37]. From 2005, the sample sizes of most relevant studies ranged from 3 to 12 [15, 17, 20, 30, 31, 38–44]. The effect of sample size on significance is related to the loading conditions and the kinematic components examined. For example, Merican and Amis reported the significant influence of ITB tension (>60 N) on patella flexion, tilt, and shift at knee flexion angle between 35° and 65°, yet had no significance at knee extension [15]. Due to the complexity of the *in vivo* loading conditions, a large sample sizes are often required to obtain reliable results. Considering both feasibility of practice and reliability of results, how many subjects should be included in a study has not been well identified.

## 3. Patella Kinematics

Normal patellar tracking has gained research attention recently. We have analyzed the studies of the past decade, explored the characteristics of the patellofemoral kinematics, and compared them with the previous conclusions.

With the aforementioned limitations, consensus has not been reached. Without regard to the nondeterminacy of the factors, we generalized and analyzed recent study results to evaluate some parameters and compare them with the previous conclusions.

The advanced search of the PubMed database was used. Inputs for the search were “patellar” (all fields) and “tracking” (all fields). The date range for publications was limited from 2005/01/01 to 2018/02/25. A total of 366 articles were searched. We singled out 14 studies, including concrete data of normal patellofemoral tracking, with the sample sizes ranging from 1 to 40 [15, 17, 20, 30–32, 37–44]. The patellar zero position is defined as the original status of patellar movement [47]. Two of the 14 studies were excluded because their patellar zero positions were not presented [17, 20]. The data collection method and coordinate system of the 12 articles were listed (Tables 1 and 2). Furthermore, patellar FHA is also an important characteristic of the patellofemoral kinematics. Researches of patellar FHA were also discussed.

**Figure 2:**
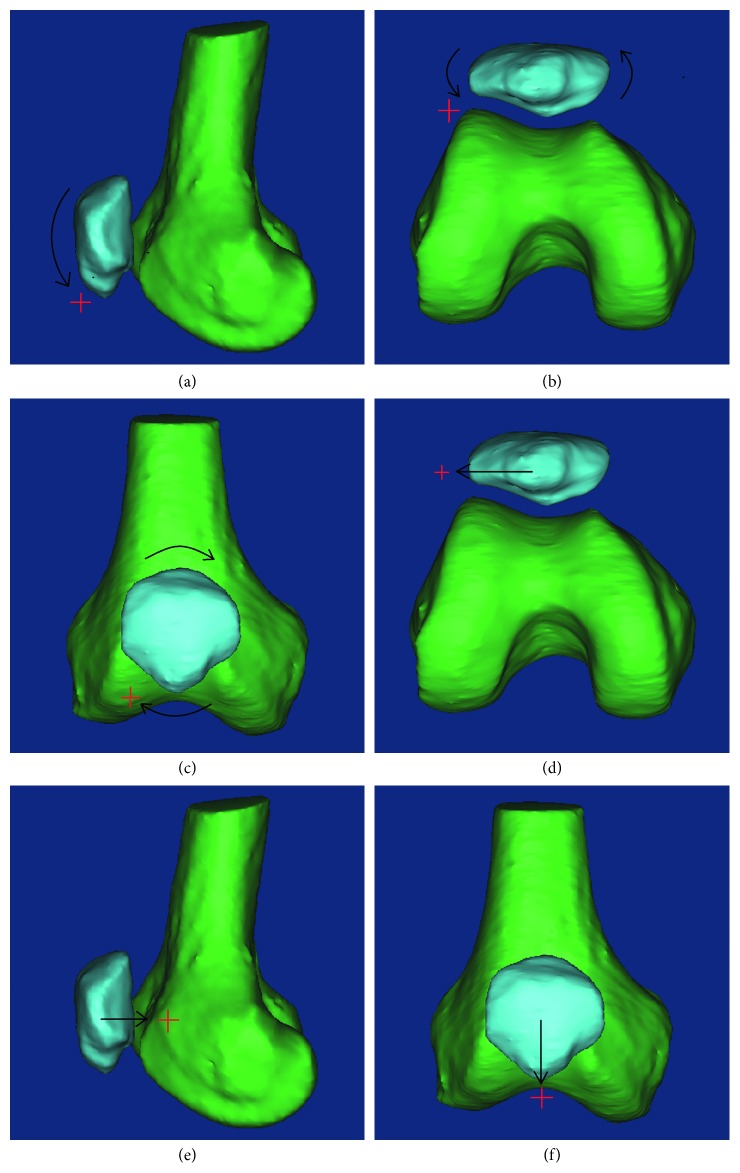
Six DOFs of patellar tracking (right knee). As the knee flexes and extends, six DOFs are involved in patellar kinematics. These are (a) flexion, (b) tilt, (c) rotation, (d) medial-lateral shift, (e) anterior-posterior translation, and (f) proximal-distal translation. Of the six DOFs, the first four indices, which are detailed in the most correlational studies, are closely related to clinical applications. In terms of DOF classification, the first three DOFs belong to the rotation parameters expressed as angles, and the last three belong to the translation parameters expressed as distance.

Knee flexion angle has been used as a reference to describe the 6 DOF movement of patella in most studies. Knee flexion angle was defined as the angle between the longitudinal axis of the tibial shaft and the femoral shaft [30]. Six DOF movement of patella are flexion (Figure 2(a)), tilt (Figure 2(b)), rotation (Figure 2(c)), medial-lateral shift (Figure 2(d)), anterior-posterior translation (Figure 2(e)), and proximal-distal translation (Figure 2(f)) [19, 28, 30, 31, 35, 36, 39–42, 44, 47, 52, 53]. Of the 6 DOFs, the first four indices, which are detailed in the most correlational studies, are closely related to clinical applications. Previous studies have shown the difference in patellar tracking among individuals; however, the difference has not been quantitatively verified given the small sample sizes [31].

**Figure 3:**
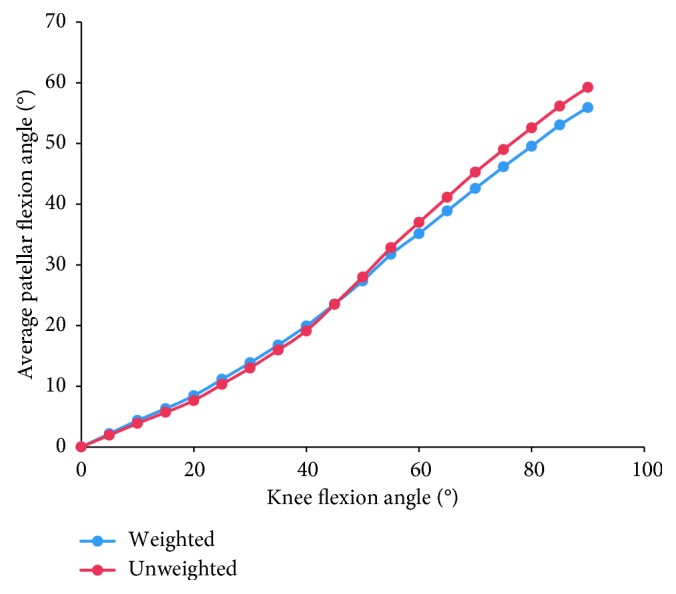
The change tendency of average patellar flexion angle with knee flexion. Considering the evident difference in sample sizes among studies, we calculated the weighted average of the patellar tracking based on the number of subjects (blue curve), as well as the unweighted average of the patellar tracking (red curve). Studies with knee flexion above 90° are included.

**Figure 4 fig4:**
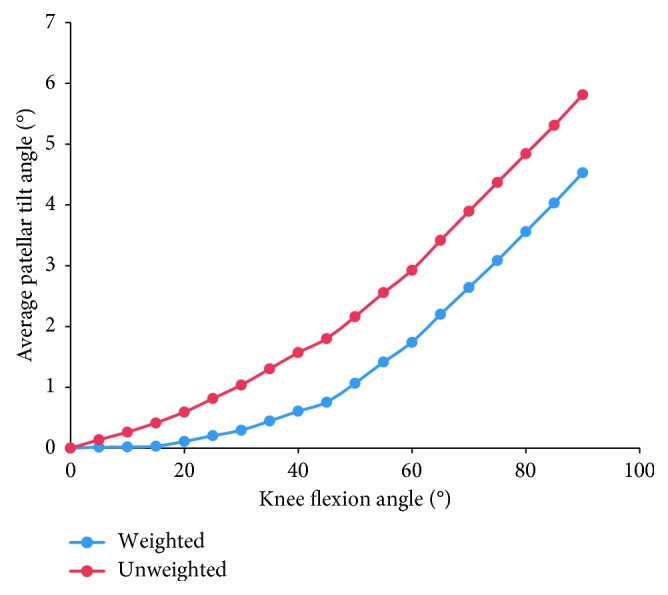
The change tendency of average patellar tilt angle with knee flexion. Blue curve is the weighted average of the patellar tracking (based on the number of subjects); red curve is the unweighted average. Studies with knee flexion above 90° are included.

**Figure 5 fig5:**
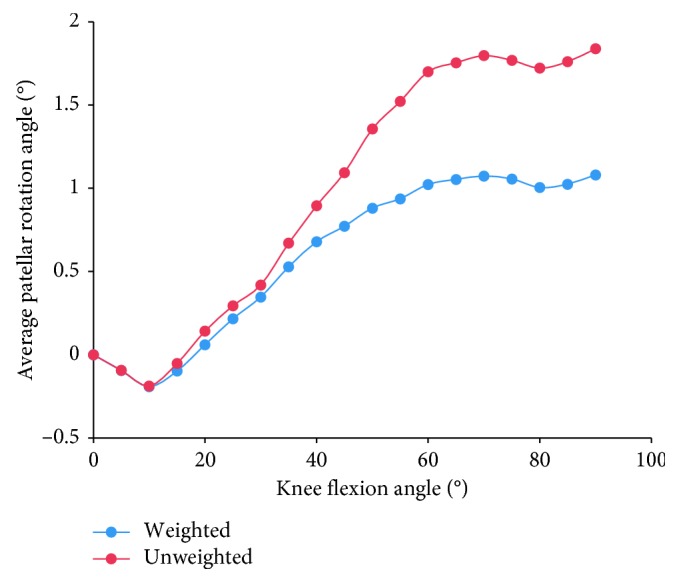
The change tendency of average patellar rotation angle with knee flexion. Blue curve is the weighted average of the patellar tracking (based on the number of subjects); red curve is the unweighted average. Studies with knee flexion above 90° are included.

**Figure 6:**
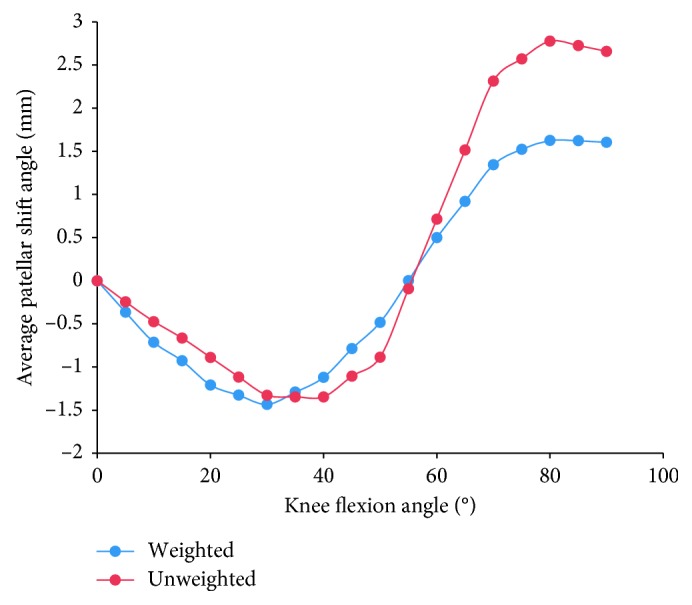
The change tendency of average patellar shift angle with knee flexion. Blue curve is the weighted average of the patellar tracking (based on the number of subjects); red curve is the unweighted average. Studies with knee flexion above 90° are included.

Furthermore, considering the evident difference in sample sizes among studies, we calculated the weighted average of the patellar tracking based on the number of subjects (blue curves in Figures 3 [Fig fig4] [Fig fig5]–6), as well as the unweighted average of the patellar tracking (red curves in Figures 3 [Fig fig4] [Fig fig5]–6). It is important to note that because of different research methods, especially coordinate system establishment, the averages calculated here are not statistically significant findings. They are meant to show trends in patellar tracking only. Therefore, the variance was also not presented. Moreover, the results of two studies varied significantly from other studies, and this could be attributed to differences in measurement methods or coordinate systems [38, 44].

### 3.1. Patellar Zero Position

We defined the patellar zero position as the original status of patellar movement [47]. A few reports selected 90° knee extension as the zero position [16], whereas most studies selected the patellar location of full or 0° knee extension as the zero position [15, 19, 32, 36, 37, 39, 40, 42, 54]. All of the 12 studies in our analysis included data relating to 0° knee extension. However, because of the different coordinate systems or zero positions, some patellar tracking components were not 0 at 0° knee extension. In order to facilitate the comparison of different studies, we used the patellar tracking components at zero position as the baseline and analyzed their trends on this basis.

### 3.2. Patellar Flexion

**Figure 7:**
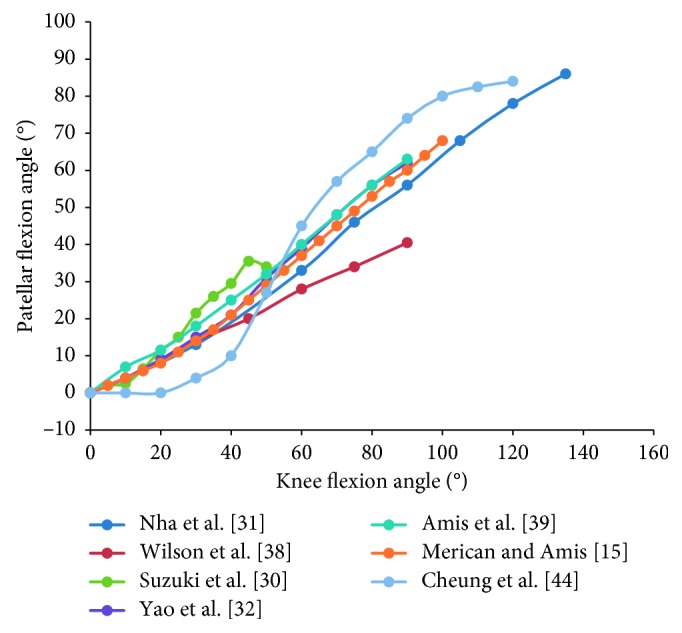
The change of patellar flexion angles with knee flexion in 12 studies. Twelve curves of different colors indicate the patellar flexion angles over knee flexion angle in 12 studies. All of the patellar flexion angles increased at a similar rate (60%–70%) with knee flexion angle.

Patellar flexion was defined as the rotation of the patella around the ML axis of the femur or patella. TEA or the line that was aligned with the most posterior points of the femoral condyles has been considered as the axis of flexion in previous studies [15, 30–32, 37–40, 44]. The positive movement was defined as the distal patella rotated backwards with respect to the proximal patella. In all of the 12 studies, the patella flexed in a positive pattern [15, 30–32, 37–44]. Moreover, the range and the change rate of patellar flexion lagged behind the knee joint flexion. As the knee flexed, the patella flexed at 60–70% times the tibiofemoral flexion angle [30–32, 39, 44] (Figure 7). The hysteresis effect was more evident at >100° knee flexion [44]. This phenomenon is about 10% different from previous review reports [36, 47]. This difference could be due to the definition of tibiofemoral flexion angles. A few researches showed that the change rate of patellar flexion could exceed that of knee flexion within a short time; however, its overall trend still falls behind the latter [44]. According to the present estimation, the lag effect manifests most evidently in the initial period (Figure 3), possibly because the patella is kept almost static at the beginning of knee flexion with a relatively slack quadriceps and patellar tendon condition.

### 3.3. Patellar Tilt

Patellar tilt was defined as the rotation of the patella around the longitudinal axis of the femur or patella. The lateral tilt (positive tilt) was defined as the movement of the patellar lateral border toward the femur with respect to the medial border.

**Figure 8:**
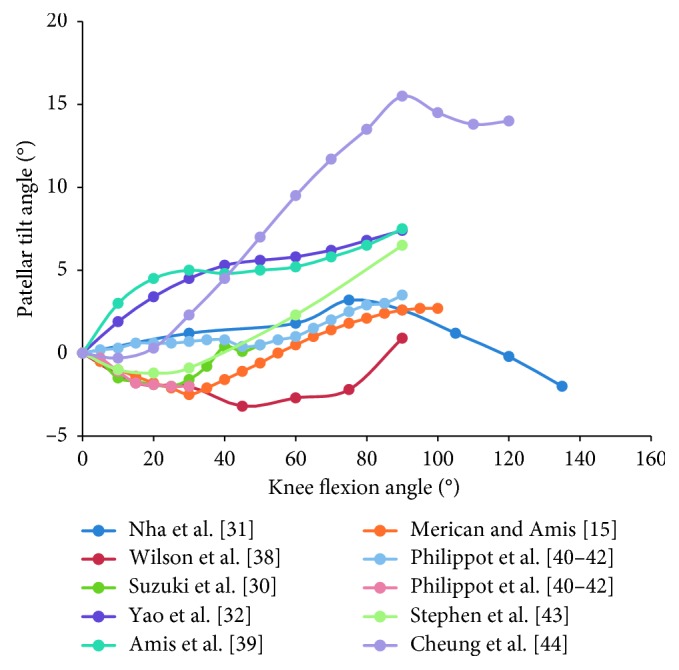
The change patellar tilt angle with knee flexion in 10 studies. Ten curves of different colors indicate the patellar tilt angles over knee flexion angle in 10 studies. Eight curves contain kinematic information from 0° to 90° of knee flexion; curves of Merican and Amis [15], Wilson et al. [38], Stephen et al. [43], and Cheung et al. [44] decreased by 1°–3° and then increased by 1°–15.5°; while curves of Nha et al. [31], Yao et al. [32], Amis et al. [39], and Philippot et al. [40–42] increased from the full knee extension. Curves of Nha et al. [31] and Cheung et al. [44] tend to decrease after knee flexion exceeds 90°.

The pattern of the patellar tilt is not significant. Within 0° to 90° of knee flexion, some studies reported that the patella tilted medially by 1°–3° and then tilted laterally by 1°–15.5° [15, 38, 43, 44], while some other studies stated that the patellar tilt occurred from the full knee extension [31, 32, 39, 40] (Figure 8). After knee flexion exceeds 90°, the patella tended to tilt medially according to a small number of studies [31, 44]. Furthermore, conclusions of different studies vary significantly about when the patella tilts fastest and when the patella returns to the zero position (the original status) of the patellar tilt. The average data imply that the patella generally tilted laterally at knee flexion angles between 0° and 90° [15, 31, 32, 38–40, 43, 44] (Figure 4).

### 3.4. Patellar Rotation

**Figure 9:**
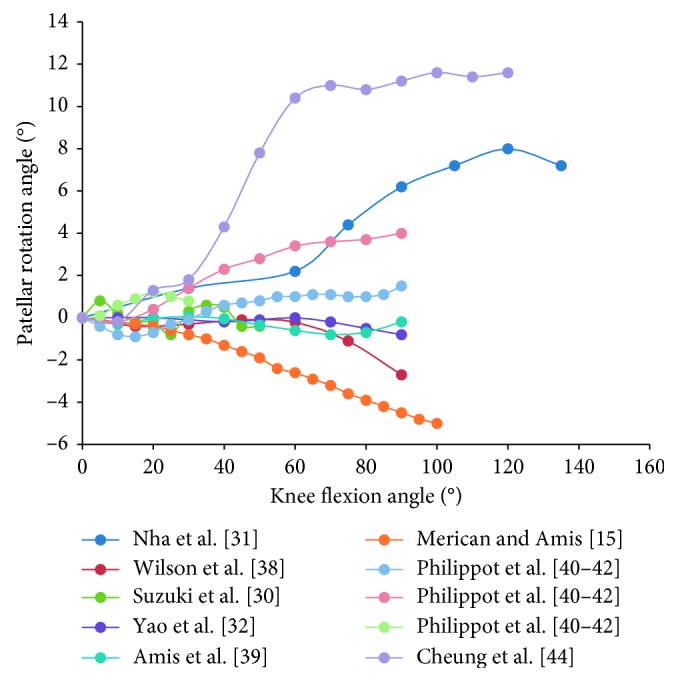
The change of patellar rotation angle with knee flexion in 10 studies. Ten curves of different colors indicate the patellar tilt angles over knee flexion angle in 10 studies. Within 30° of knee flexion, all curves are confined to the range from −1° to 2°. After knee flexion exceeds 80°, curves of Nha et al. [31], Cheung et al. [44], and Philippot et al. [40–42] increased to greater than 2°, while curves of Merican and Amis [15] and Wilson et al. [38] decreased to −2°.

Patellar rotation is described as the movement of the patella around the AP axis of the femur or patella. Lateral rotation (positive rotation) was defined as the outwards rotation of the distal patella with respect to the proximal patella. Based on data from ten studies, we found that the patellar rotation angle was confined to the range from −1° to 2° within 30° of knee flexion [15, 30–32, 38–42, 44]. Some studies further reported that the patella rotated medially by 0.2°–0.9° and subsequently showed persistent lateral rotation or fluctuant medial-lateral rotation [32, 38–41, 44]. The initial transformation from medial to lateral rotation occurred at 10°–15° knee flexion [38–41, 44] (Figure 9). Moreover, other studies showed that the patella rotated medially [15] or laterally [31] with an overall movement range fluctuating from 5° medial rotation to 11° lateral rotation [15, 31, 32, 38–41, 44]. The average curve showed that the patella rotated slightly medially at the beginning of flexion (<10°) before its long-term lateral rotation with transient fluctuation (Figure 5).

### 3.5. Patellar Shift

**Figure 10:**
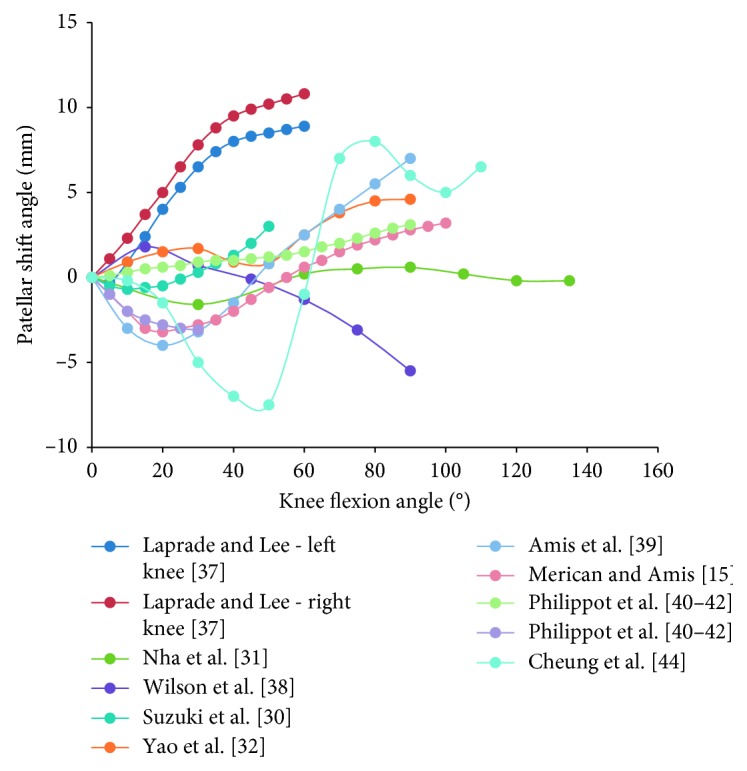
The change patellar shift angle with knee flexion in 11 studies. Eleven curves of different colors indicate the patellar shifts over knee flexion angle in 11 studies. Curves of Merican and Amis [15], Suzuki et al. [30], Nha et al. [31], Amis et al. [39], Philippot et al. [40–42], and Cheung et al. [44] decrease first and then increase, while curves of Wilson et al. [38] increases first and then decreases.

Patellar shift, sometimes called glide, was defined as the transverse displacement of the patellar center point along the ML axis of the femur or patella. Lateral shift was defined as positive movement. Most studies revealed that the patella shifted medially prior to lateral translation [15, 30, 31, 39, 42, 44], which occurred at 10°–30° knee flexion in the majority of cases [15, 30, 31, 39, 42] (Figure 10). This finding is consistent with other reviews [36]. However, some other studies reported that the patella shifted laterally with possible temporary medial shift [32, 37, 40]. By contrast, Wilson et al. [38] reported that the patella translated medially (5 mm medial to the patella zero position) after a slight lateral shift at the initial stage of flexion. Furthermore, the averaged data showed that the patella manifested a medial shift before lateral translation and tended to shift medially again at the later flexion stage (>80°) (Figure 6). However, since there are few studies considering deep knee flexion (>90°), no agreement on patellar tracking at the end piece of knee flexion has been established.

In addition, due to the absence of guidance values of anterior-posterior and proximal-distal translation of the patella for clinical diagnosis and management [47], corresponding research about these two DOFs is scarce.

### 3.6. Patellar Finite Helical Axis

**Figure 11:**
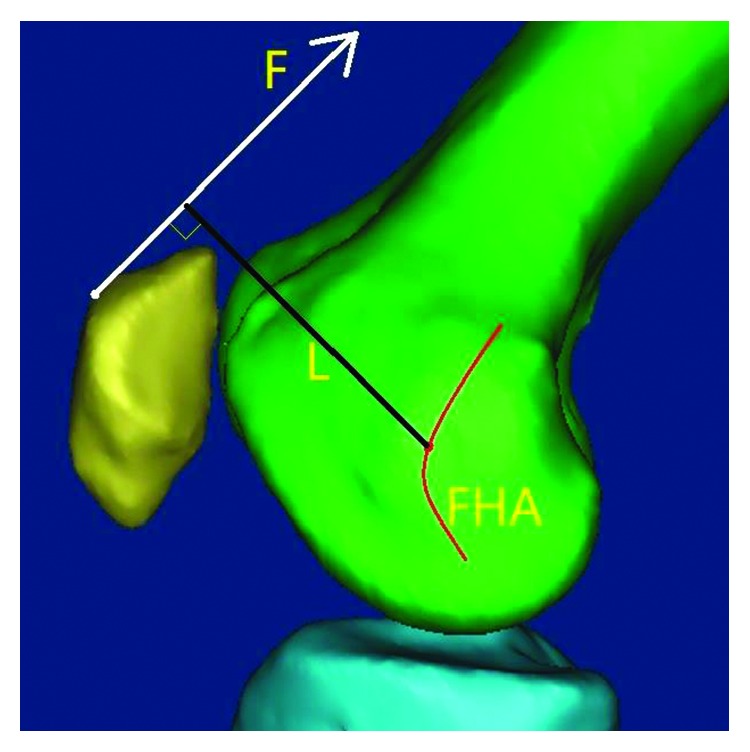
Patellofemoral arm and moment of force during flexion or extension. Patellofemoral arm of force is defined as the distance from the resultant force of the quadriceps to FHA. Patellofemoral moment of force is defined as the product of the arm of force and the resultant force of quadriceps. *F*: resultant force of quadriceps, FHA: patellar finite helical axis, *L*: arm of resultant force of quadriceps, and *M*: moment of resultant force of quadriceps (*F* × *L*).

Some surgeons have established a suite of surgical procedures to treat patellofemoral disorders and put them into practice. These procedures include medial patellar reticulum reefing, lateral patellar reticulum release, and tibial tubercle transposition. These operations are effective to some extent. However, patients tend to experience patellofemoral disorders again some time later [55], because the alignment relationship between the trochlea and patella, which has been corrected during the aforementioned operations, is a partial manifestation of patellofemoral biomechanics. The arm and moment of quadriceps force, which remains uncorrected, might play an important role in joint motion. The quadriceps arm could be defined as the distance from the quadriceps to FHA. Quadriceps moment could be defined as the product of the arm and the force (Figure 11). Thus, some scholars started diverting their attention to FHA. Coughlin et al. [56] revealed that the patella presented a circular motion around the TEA with a 30.6 mm diameter from 0° to 90° flexion. Amis et al. [39] reported that FHAs were a group of dynamic axes near the posterior condyles by means of a “floating axis” system. However, these previous experiments only raised a vague position of FHA instead of a quantitative analysis. Yao et al. [32] proposed a method of calculating the FHAs with the MR images and compared its relationships with TEA. Despite the finitude of studies about FHA, scholars have agreed that normal FHA fluctuated around TEA or the posterior condylar axis. Further studies on the relationship between FHA and PFPS are warranted.

## 4. Factors Affecting Patellofemoral Kinematics

Numerous factors can affect patellofemoral kinematics, including trochlear groove morphology, muscular and retinacular stretch, and tibial rotation [17, 36, 39, 41, 53]. At the initial period of flexion, soft tissues (quadriceps, patellar tendon, and medial and lateral retinaculum) play a vital role in patellar tracking. During further flexion, the status of the patella is determined by the morphology of the trochlear groove and patellar facet [53].

### 4.1. Spatial Structure of Patellofemoral Facets

Femoral trochlear groove is defined as the sulcus in the anterior junction of the bilateral condyles. Because the relationship between the patella and trochlear groove could be clearly identified in clinical images, it has been widely used in clinical diagnosis. At full knee extension, the patella is not congruent with the femoral trochlea. As the knee flexes, the patella enters into the groove, shifts medially, then laterally [53]. *In vivo* study has discovered the significant correlation between the sulcus morphology and patellar shift and tilt [57]. A flat lateral facet of the trochlear groove may increase the risk of patella lateral subluxation or dislocation. Wang et al. further reported that the angle between the PVR and the trochlear groove slightly changed during normal knee flexion [24], which may provide an important role in the stabilization of patellofemoral kinematics.

### 4.2. Soft Tissue Balancing

Besides the osseous structures, the effects of soft tissues (quadriceps and ITB) on patellar kinematics as previously mentioned have been investigated widely. Despite some differences, scholars have reached a consensus that the quadriceps, especially the vastus medialis and vastus lateralis, have a greater influence on patellar tracking [17]. Accordingly, when performing knee arthroplasty, surgeons often adopt quadriceps balancing as a crucial measure to perfect patellofemoral kinematics. Before surgery is performed, other therapeutic means can be applied to improve the patellar tracking. By comparing the efficacy of injecting botulinum toxin type A (BoNT-A) and placebo to the vastus lateralis muscle for the patients with chronic anterior knee pain (AKP) associated with quadriceps muscle imbalance, Singer et al. [58] concluded that BoNT-A injection produced a greater reduction in pain and disability than placebo injection. This conclusion has provided preliminary support for the role of BoNT-A as a promising adjunct to nonsurgical management of individuals with chronic AKP [59].

### 4.3. Interaction between Patellofemoral and Tibiofemoral Joints

It has been reported that tibial rotation and varus, as well as valgus, can influence patellar tracking [20, 36, 54]. As the tibia rotates medially with respect to the femur in the initial stage of flexion, the patella tends to move to the lateral side toward the tibial tubercle [36]. *In vitro* and *in vivo* studies demonstrated that tibia medially rotation could cause patellar lateral shift [18, 20]. Hence, tibial rotation should be taken into account when investigating patellar tracking. Meanwhile, quadriceps force is transmitted to the tibiofemoral joint through the patellofemoral joint. However, while tibiofemoral joint replacement has gained more attention, its correlation with patellofemoral biomechanics has not yet been fully established.

## 5. Prospect

With a high morbidity of 13–20%, PFPS severely affects the quality of life of patients. Previous studies showed that kinematic characteristics of the patellofemoral joint are closely related to PFPS. However, normal patellar kinematics remains undefined. Analyzing the various research methods and their results, we conclude that during knee flexion, the patella flexes but lags behind the tibiofemoral joint (30–40%). Most scholars reported a medial patellar tilt, rotate, and shift in the initial stage of knee flexion and a lateral tilt, rotation, and shift thereafter. Moreover, a few studies indicated that the FHA fluctuates near the TEA or posterior condylar line. These kinematic characteristics can provide clues for understanding the normal patellar tracking and distinguish different kinds of maltrackings.

The approaches taken in studies in the literature are different. Therefore, reaching a consensus with the current results is difficult. As normal patellar biomechanics has been widely regarded as the basis and a condition to keep the knee from disorders, further investigations based on objective and uniform methods, as well as larger samples, are needed. With the rapid advancement of technology, measurement techniques suitable for complex physiological activities need to be developed. Furthermore, patellofemoral replacement and patellar arthroplasty are both currently performed in the supine position without any load, which could not ensure an equal tracking of the patella when the patients stand up after surgery. More reasonable design ideas are needed to address the discrepancy between the results of the current scientific research and clinical application.

## Figures and Tables

**Table 1:** Study methods of normal patellar tracking (from 2005).

References	Size	*In vivo/in vitro*	Bear loading	Quadriceps tension (*N*)	Knee flexion/extension	ROM of knee	Dynamic/static	Acquisition methods
Laprade and Lee [37]	40 bilateral	*In vivo*	Full	—	Flexion	0°–60°	Dynamic	Reception-transmission device
Nha et al. [31]	8	*In vivo*	Full	—		0°–135°	Static	MRI and dual-orthogonal fluoroscopic system
Wilson et al. [38]	10	*In vivo*	Full	—	Flexion-squatting	0°–90°	Static	Specific device
Suzuki et al. [30]	12	*In vivo*	Full	—	Extension-going upstairs	0°–50°	Static	MRI
Yao et al. [32]	1	*In vivo*	0	—	Flexion	0°–90°	Static	MRI
Amis et al. [39]	8	*In vivo*	—	175	Flexion + extension	0°–90°	Dynamic	Reception-transmission device
Merican and Amis [15]	9	*In vivo*	—	175	Extension	0°–100°	Dynamic	Specific device
Philippot et al. [40]	6	*In vivo*	—	10	Flexion	0°–90°	Dynamic	Motion analysis system
Philippot et al. [41]	9	*In vivo*	—	10	Flexion + extension	0°–90°	Dynamic	Motion analysis system
Philippot et al. [42]	6	*In vivo*	—	10	Flexion + extension	Full extension to full flexion	Dynamic	Motion analysis system
Stephen et al. [43]	8	*In vivo*	—	175	Flexion	0°–90°	Static	Specific device
Cheung et al. [44]^a^	3	*In vivo*	—	0	Flexion + extension	0°–Full flexion	Static	Reception-transmission device

^a^This study was performed on complete cadavers without stretching quadriceps. We set the greatest range of motion (ROM) to 120° to match it with our analysis. Furthermore, bone probe and skin receptor results were both adopted, and we selected the results based on the former.

**Table 2:** Reference axes or measurement methods of normal patellar tracking (from 2005).

References	Reference axes of shift	Reference axes or measurement methods of flexion	Reference axes or measurement methods of tilt	Reference axes or measurement methods of rotation
Laprade and Lee [37]	TEA	—	—	—
Nha et al. [31]	TEA	TEA	Longitudinal axis of the patella	AP axis of the patella
Wilson et al. [38]	TEA	TEA	Longitudinal axis of the patella	Floating axis perpendicular to the first two axes
Suzuki et al. [30]	TEA	The angle between the superior-inferior axis of the patella and long axis of the femur projected onto the sagittal plane of the femur	The angle between the ML axis of the patella and the TEA projected onto the transverse plane of the femur	The angle between the ML axis of the patella and the TEA of the femur projected onto the coronal plane of the femur
Yao et al. [32]	TEA	TEA	Longitudinal axis of the patella	AP axis of the patella
Amis et al. [39]	Axis perpendicular to the long axis of the femoral shaft and parallel to the plane containing the most posterior points of the femoral condyles	Same to the axis of shift	Longitudinal axis of the patella	Floating axis perpendicular to the first two axes
Merican and Amis [15]	Axis aligned with the most posterior points of the femoral condyles	Same to the axis of shift	Longitudinal axis of the patella	AP axis crossing geometric center of the patella
Philippot et al. [40]	TEA	—	Longitudinal axis of the patella	AP axis of the patella
Philippot et al. [41]	—	—	—	AP axis of the patella
Philippot et al. [42]	ML axis of patella	—	Longitudinal axis of the patella	AP axis of the patella
Stephen et al. [43]	—	—	Longitudinal axis of the patella	—
Cheung et al. [44]	TEA	TEA	The line joining the greater trochanter and midpoint between two femoral epicondyles	Cross product of the first two axes

## Supplementary Materials

Supplementary Materials The diagrams of three primary coordinate systems are femoral coordinate system, patellar coordinate system, and femur-patella hybrid coordinate system.
