# Supplementary material for: Research Methods and Progress of Patellofemoral Joint Kinematics: A Review
Source: J Healthc Eng. 2019 Mar 24;2019:9159267. doi: 10.1155/2019/9159267 (PMC6451817; doi:10.1155/2019/9159267)
Supplement: Supplementary Materials — The diagrams of three primary coordinate systems are femoral coordinate system, patellar coordinate system, and femur-patella hybrid coordinate system. [file 9159267.f1.pdf]

Supplementary material 1

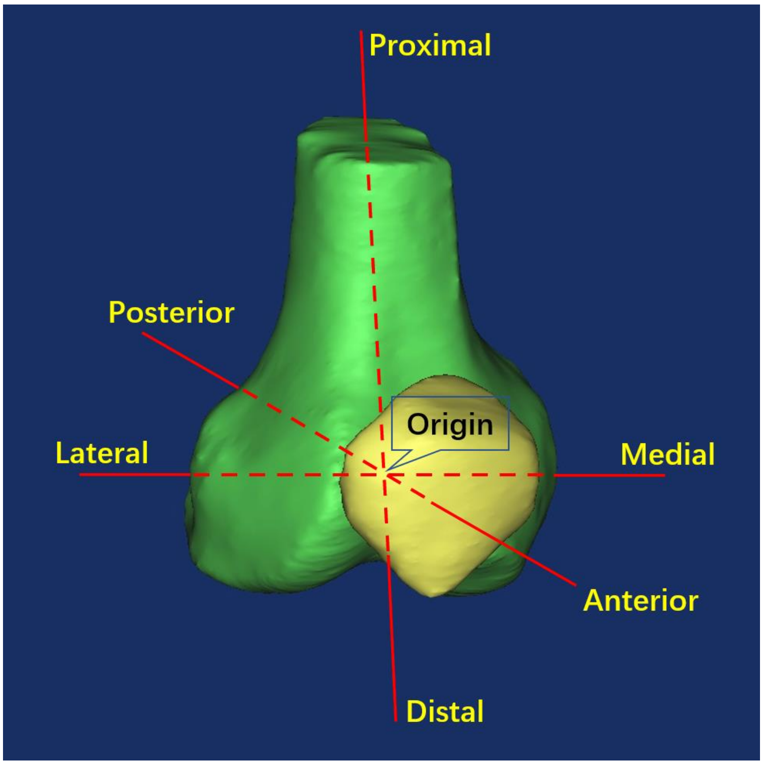

a. Femoral coordinate system

The three axes in the femoral coordinate system include the proximal-distal (PD) axis (longitudinal axis), medial-lateral (ML) axis, and anterior-posterior (AP) axis. Mostly, the femoral anatomical and mechanical axes were used to determine the PD axis, and the TEA was regarded as ML axis. The femoral AP axis could be established by the cross product of the PD and ML axes. And the midpoint of the TEA was defined as the origin.

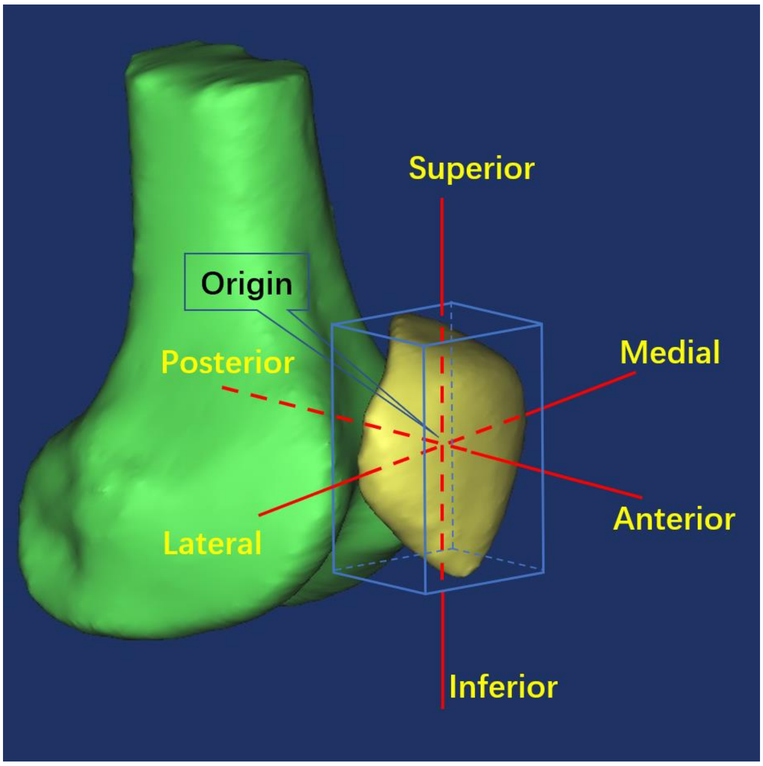

b. Patellar coordinate system

A fitting bounding box (FBB) was established around the patella, which touched the superior-inferior, anterior-posterior, and medial-lateral borders of the patella. The center of the FBB was defined as the origin of patellar coordinate system.

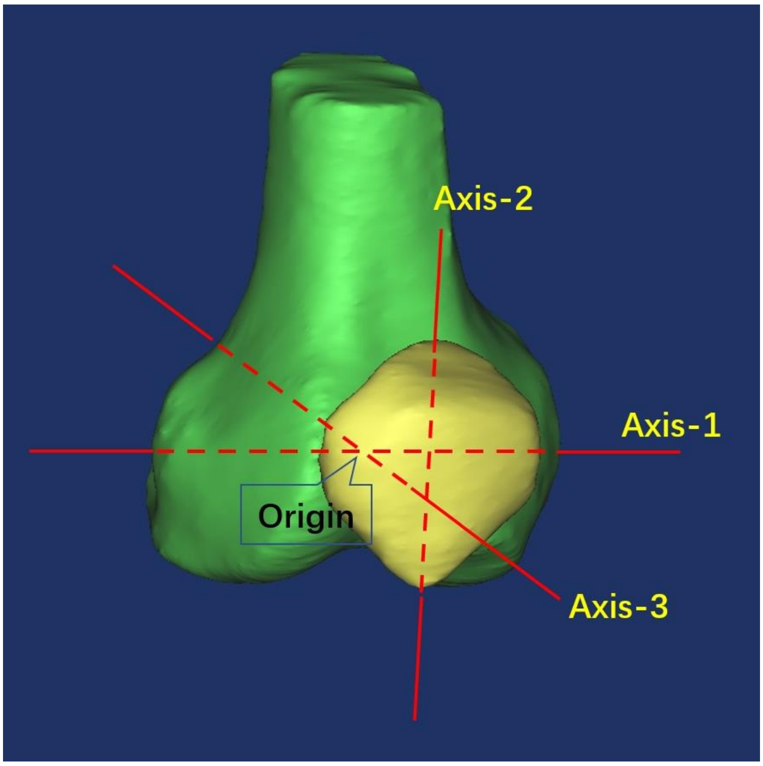

c. Femur-patella hybrid coordinate system

Axis-1 is defined as the femoral ML axis. Axis-2 is defined as the patella PD axis and moved with patella. Axis-3 is defined as the axis perpendicular to Axis-1 and Axis-2. The midpoint of the Axis-1 could be selected as the origin.
